# When AI ethicists shoot from the hip: Stone-Age moral psychology in the age of AI

**DOI:** 10.3389/fpsyg.2026.1813445

**Published:** 2026-06-18

**Authors:** Tomislav Bracanović

**Affiliations:** Institute of Philosophy, Zagreb, Croatia

**Keywords:** AI ethics, ethical expertise, evolutionary psychology, overdetection of agency, overmoralization of affectively charged situations, overpropagation of morally salient information, professional self-interest

## Abstract

AI ethics has expanded rapidly, with a substantial share of its scholarly output arguably treating a wider-than-warranted range of AI-related developments as pressing moral problems. The paper argues that this can, to some extent, be explained by three evolved cognitive mechanisms: the overdetection of agency, the overmoralization of affectively charged situations, and the overpropagation of morally salient information. It further suggests that these mechanisms are likely at work among AI ethicists under conditions particularly conducive to their activation, including the genuine novelty of many AI technologies and the moral questions they raise, the limited protective effect of ethical expertise against certain cognitive and moral biases, and a professional environment that rewards the identification of ever-new moral problems. The paper develops a conceptual framework comprising a set of arguments and explanatory hypotheses aimed at explaining why some AI-related concerns proliferate and intensify in ways disproportionate to the actual risks and capabilities of existing AI systems.

## 1 Introduction

In 1991, the prominent American ethicist Rachels (1991) published an essay aptly titled “When philosophers shoot from the hip.” He discussed a widely publicized case in which several ethicists criticized a couple's decision to conceive a child to obtain compatible bone-marrow tissue to save their dying daughter, describing the decision as morally outrageous and accusing the parents of treating the newborn child merely as a means. Rachels's analysis showed that these reactions relied on a hasty invocation of ethical principles, combined with a striking insensitivity to real-life contexts and flesh-and-blood human predicaments. The practice at issue (now commonly referred to as “savior siblings”) has, in the meantime, become rather uncontroversial, and Rachels's conclusion has largely been vindicated: ethicists often, when responding to novel and emotionally charged cases, are trigger-happy in their judgments, reinforcing prevailing moral anxieties rather than subjecting them to clearheaded critical scrutiny.

The central thesis of this paper is that a substantial segment of the scholarly output within the field of AI ethics—understood here as a discipline concerned with the ethical aspects of AI and its societal implications—can be characterized as “shooting from the hip.” More specifically, AI ethics seems to treat a wider-than-warranted range of AI-related developments as pressing moral problems, and it is suggested that this may result from three cognitive mechanisms well documented in evolutionary psychology and related disciplines: the overdetection of agency, the overmoralization of affectively charged situations, and the overpropagation of morally salient information. As further argued, the operation of these mechanisms is amplified by three factors specific to the field of AI ethics: the genuine novelty of many AI technologies and the moral questions they raise, the limited protective effect of ethical expertise against certain cognitive and moral biases, and a professional environment that rewards the identification of ever-new moral problems.

These three cognitive mechanisms and their three amplifying factors are presented as components of a conceptual framework comprising a set of arguments and explanatory hypotheses about this specific internal dynamics of AI ethics, with the potential to inform future empirical research and more precise, testable predictions. Although similar concerns are often discussed under headings such as AI hype or anthropomorphism, the present paper seeks to address a somewhat different set of questions. While anthropomorphism can be viewed as a specific manifestation of the agency overdetection mechanism, the present approach focuses on this broader mechanism and complements it with an account of two further processes: the treatment of merely affectively charged situations as genuine moral problems and the spread of such overmoralized information. Similarly, discussions of AI hype, understood as exaggerated expectations and fears about AI capabilities and impacts, typically remain largely descriptive, whereas the proposed framework seeks to unpack such patterns into more basic components and to uncover their evolutionary underpinnings. It should be emphasized, however, that none of this is meant to deny that many concerns in AI ethics are legitimate and important, but rather to suggest that, alongside such valid concerns, there are likely also excessive responses that may be partly explained in evolutionary-psychological terms.

Section 2 outlines the core assumptions of evolutionary psychology and explains their relevance for ethical theorizing. Section 3 introduces three cognitive mechanisms, or “triggers,” that contribute to disproportionate ethical assessments of AI: the overdetection of agency, the overmoralization of affectively charged situations, and the overpropagation of morally salient information. Section 4 examines why AI ethics may be particularly susceptible to these triggers by identifying three field-specific amplifying factors: the genuine novelty of certain AI technologies and the moral questions they raise, the limited protective effect of ethical expertise against certain cognitive and moral biases, and a professional environment that rewards the identification of ever-new ethical problems. The conclusion briefly restates the main claims and highlights the benefits of the proposed framework for both evolutionary psychology and AI ethics.

## 2 Evolutionary psychology and its relevance for ethics

In addition to its close ties to the philosophy of mind, evolutionary psychology has been recognized as relevant to ethics since its beginnings. By providing accounts of the evolutionary origins of various forms of human behavior (such as cooperation and altruism), it offers a naturalistic foundation for understanding the psychological underpinnings of moral judgment and action. For this reason, it occupies a prominent place in evolutionary ethics (James, 2011), particularly in debates about the objectivity of ethics, the sources of moral motivation, and the evolutionary constraints on moral reasoning. Evolutionary psychology has also been the subject of a philosophical debate regarding its methodological coherence and scientific status. Despite substantial criticism (e.g., Buller, 2005), it is generally regarded as representing a progressive research program (Ketelaar and Ellis, 2000).

Evolutionary psychology has two main ingredients typical of research programs. One of them is a hard core—a set of fundamental principles and assumptions shared by all researchers within the program—and the other is a protective belt—an open-ended range of auxiliary assumptions and hypotheses that apply these core principles and assumptions to specific phenomena (Lakatos, 1970). Evolutionary psychologists, from the program's early founders (Barkow et al., 1992) to contemporary textbook authors (Workman and Reader, 2021), relatively consistently identify the following principles and assumptions as its theoretical core: (a) the human mind, instantiated in the brain, is an information-processing system whose mechanisms have been shaped by natural selection, (b) it is, to some extent, made up of functionally specialized mechanisms that operate outside the scope of conscious awareness, and (c) many of these mechanisms are solutions to adaptive problems from evolutionary past and therefore may not be well suited to current conditions: they act as proximate causes of our cognitive and behavioral responses, while themselves having ultimate causes in evolutionary pressures operating in ancestral environments. Metaphorically, much of human cognition and behavior remains adapted to, and still bears traces of, Stone-Age conditions, even when applied in very different social and technological settings.

The protective belt of evolutionary psychology has gradually expanded to encompass a broad range of phenomena. While early research in evolutionary psychology focused mainly on theoretical explanations of cooperation, language, and mating strategies, later developments have increasingly shifted toward applying these insights to domains such as mental wellbeing, interpersonal relationships, education, economics, public policy, communication, media, and, most recently, AI and robotics (Crawford and Salmon, 2004; Roberts, 2016; Shackelford, 2021). The relationship between evolutionary psychology and AI is twofold: evolutionary-psychological ideas are either used by engineers and designers to incorporate certain elements of human mental architecture into machines (Evans and de Back, 2016; Finkelstein, 2021), or they are applied to examine how AI influences human cognition and behavior, with both positive and negative effects (Wilson et al., 2021). Building on this, the next section extends this relationship by suggesting that a segment of the scholarly output in AI ethics may reflect the operation of three evolved cognitive mechanisms that contribute to disproportionate assessments of various AI technologies.

## 3 AI ethics and its three evolutionary triggers

The development of AI has been accompanied by what is now commonly referred to as “AI hype,” referring to exaggerated expectations (especially fears) about the capabilities and long-term consequences of AI systems. This phenomenon can be illustrated by the rapid spread of information about the allegedly morally problematic aspects of AI. For example, at the time of completing this paper (March 2026), Google searches show similar or even higher numbers for AI-related dangers compared to longstanding sources of moral concern: “AI apocalypse” returns about 595,000 results, surpassing “nuclear apocalypse” (around 384,000); “AI takeover” returns roughly 1.18 million results, comparable to “military takeover” (around 1.3 million); and “racist AI” returns about 700,000 results, exceeding not only “racist society” (around 511,000) but also very specific historical references such as “racist apartheid” (around 161,000) or “Ku Klux Klan racism” (around 11,100). Although AI hype has itself become a subject of scholarly reflection, mainly concerning its presence in public discourse and among the general population (Placani, 2024; LaGrandeur, 2024), the aspect that has received no attention, at least to my knowledge, is that AI ethics itself may exhibit similar distortive dynamics.

A noticeable feature of the scholarly output in AI ethics appears to be its treatment of a wider-than-warranted range of AI-related developments as pressing moral problems. Of course, it is difficult to rank problems by urgency within any branch of applied ethics. However, the sense that something unusual is taking place in AI ethics has become increasingly common. For example, Müller (2020) begins his overview of AI and robotics ethics by noting that the field revolves around many concerns, many of which turn out to be “rather quaint,” “predictably wrong,” or only “moderately relevant.” Powers and Ganascia (2020) warn that “overestimation of future technological/ethical problems leads some ethicists to become (amateur) futurists, and these futurists often spend an inordinate amount of time worrying about technological problems that will never come to pass.” Königs (2025) offers similarly critical observations, suggesting that “the field has grown out of proportion” and that “just because AI ethics has presented a rather bleak view of AI's ethical implications does not mean that they really are that bleak.” One of the main reasons behind this overextension, as will be argued, is that parts of the scholarly output in AI ethics may be influenced by three proximate cognitive mechanisms whose origins can be traced back to ancestral environments. However, when triggered in new, AI-saturated environments, these mechanisms may elicit disproportionate evaluative responses. Hereafter referred to as “triggers,” these mechanisms include the overdetection of agency, the overmoralization of affectively charged situations, and the overpropagation of morally salient information (due to space constraints, each is outlined schematically and with a degree of terminological parsimony).

### 3.1 Overdetecting agency

Probably the central trigger that draws AI ethicists' attention to various AI systems is what evolutionary psychologists call an agency-detection mechanism, often referred to as the “hyperactive agent-detection device” (Barrett, 2000; Dennett, 2006). Although initially developed in discussions about the evolutionary origins of religious belief, the mechanism itself is broader in scope. An agent-detection device can be understood as a cognitive mechanism shaped by natural selection to address a recurring adaptive problem: detecting the presence of other minds (human or non-human) and deciphering their intentions under conditions of uncertainty and possible threat. The evolutionary rationale is straightforward: recognizing that someone (or something) is nearby and determining whether their intentions are friendly, hostile, or neutral could be crucial for survival and reproduction. The key point is that the costs of failing to detect a genuine agent (especially a hostile one) are usually much higher than the costs of responding to a false alarm (e.g., mistaking a shadow for a hidden intruder). As a result, selection pressures have favored agency-detection mechanisms that are overly sensitive to cues of agency, such as bilateral symmetry or seemingly goal-directed motion. These mechanisms, in other words, are biased toward overdetection and lead to the attribution of agency or mentality—ranging from goal-oriented intentionality to sentience or moral status (distinctions are omitted here for simplicity)—even when such properties are not actually present.

Contemporary AI systems are not agents in any meaningful sense of the word (they lack intentions, emotions, or desires), but they are especially good at triggering our evolved agency-detection mechanism. Embodied AI, such as robotic systems, strongly activate this mechanism because they are often designed to display cues such as human- or animal-like appearances, autonomous movement, responsiveness to environmental conditions, and interactive feedback. The detection of agency extends beyond robotic devices. AI expert systems and conversational agents, whose “embodiment” may be limited to text on a screen, can evoke similarly powerful impressions of interacting with entities endowed with mental states roughly similar to our own. As Liddle and Shackelford (2021) note, a hyperactive agency-detection device causes us to interpret ambiguous stimuli *as agents*, but it also leads us to see them *as the results of agents*. It is not surprising, therefore, that interactions with conversational AI systems can lead to emotional attachment or even dependence, to the extent that chatbots are perceived as confidants or advisors (Gao and Mvondo, 2025).

There are no studies specifically examining AI ethicists' susceptibility to agency overdetection. However, there are several strong *prima facie* reasons to believe they are just as vulnerable to this temptation as anyone else (see also section 4). First, by the very nature of their work, AI ethicists are more exposed to information about AI systems than the general public is. Their professional environment exposes them not only to technical descriptions or research papers but also, often just a click away, to images and demonstrations of AI technologies. Many of them actively use such systems in their own work, especially chatbots, large language models, and automated translation tools. Furthermore, ethicists—like philosophers in general—are, by training, receptive to new, counterintuitive, and conceptually provocative ideas and thought experiments. The idea of artificially created intelligent agents, including “artificial moral agents,” naturally fits within this class of philosophically appealing concepts, for which significant conceptual groundwork has already been established in discussions of machine ethics (Anderson and Anderson, 2011). Since AI ethicists approach these issues with a conceptual inventory already primed to attribute agency, they may be particularly likely to ascribe agency to AI systems.

Although the above hypothesis cannot be supported with concrete data, a fair amount of circumstantial evidence suggests it is plausible. Across academic articles, monographs, and conference calls in AI ethics, AI systems are frequently described with mentalistic language that assigns them beliefs, intentions, understanding, agency, or responsibility. This tendency is even more noticeable in public-facing activities, such as popular lectures, interviews, and opinion pieces, which also play a significant role in shaping the standards of engagement with AI systems within the AI ethics community. It is particularly indicative that similar patterns also appear among AI and robotics experts themselves. It is well documented (Turkle, 2011) that laboratory interactions with AI systems and social robots often trigger strong emotional responses—including concern, discomfort, or moral unease—even among experienced researchers. Two well-known examples, frequently discussed in the literature, help illustrate this point: the primitive chatbot ELIZA, created in 1966 by Joseph Weizenbaum, elicited strong beliefs in its users about its intelligence and empathy; and in 2022, one of Google's engineers gained significant public attention for claiming that the chatbot LaMDA had achieved a form of understanding or awareness. In a nutshell, if agency overdetection (here understood in an inclusive sense to encompass sentience and moral status) is a recognized risk even among engineers and designers who build these systems, there is little reason to assume that AI ethicists are immune to the same cognitive bias.

### 3.2 Overmoralizing affectively charged situations

The second trigger most likely contributing to a noticeable trend in AI ethics of treating a wider-than-warranted range of AI-related developments as pressing moral problems is an evolved psychological tendency to overmoralize situations that are merely affectively charged. The idea is that our cognition includes specialized mechanisms that regulate cooperation and social exchange, focusing on the quick detection of situations involving potential norm violations. Since false negatives (mistaking a cheater for a cooperator) are more costly than false positives (mistaking a cooperator for a cheater), these mechanisms are biased toward heightened readiness to treat perceived irregularities as morally relevant (Cosmides, 1989; Cosmides and Tooby, 2008). Jonathan Haidt's social intuitionist model expands on this idea by illustrating that human moral judgment is typically driven by fast, affect-laden intuitions, with more deliberate moral reasoning coming into play later, mainly to justify initial reactions (Haidt, 2001; Haidt and Bjorklund, 2008). As experimental studies have demonstrated, people often judge emotionally charged but harmless scenarios (such as carefully constructed violations of dietary or sexual taboos) as morally wrong, even though they cannot provide clear reasons for their condemnation. Moral reasoning in such cases, as Haidt (2001) puts it, functions less like a judge or scientist seeking the truth and more like a lawyer defending a client, constructing *post hoc* rationalizations that only create the appearance of reflective deliberation. Pinker's (2014) convenient metaphor for this phenomenon is the “moralization switch,” understood as a psychological process that is selectively but largely unconsciously activated, transforming matters of “preference and prudence” into matters of “sin and virtue.”

Demonstrating that a degree of overmoralization occurs among AI ethicists is far from straightforward and cannot be addressed in depth within the limited space available here. However, there are indirect ways to support the conjecture that a systematic misfiring of their “moralization switch” may be operating along three dimensions.

The first dimension can be characterized in terms of scope, that is, the tendency to treat too many issues as moral problems. For example, there is a remarkably broad array of topics commonly included under the umbrella of “AI ethics.” In contemporary reference literature (e.g., Dubber et al., 2020; Gunkel, 2024), AI is described as “disruptive” across numerous fields, including employment, healthcare, transportation, education, governance, social services, law, criminal justice, entertainment, communication, politics, and warfare, with consequences for a plethora of sensitive issues, including privacy, security, autonomy, transparency, discrimination, democracy, responsibility, equality, race, gender, sexuality, identity, dignity, creativity, diversity, wellbeing, human rights, LGBTQ rights, and even animal rights (both lists could be expanded). This is not to deny that many of these issues are important. Rather, the point is that they are unlikely to all be equally morally pressing, especially given that earlier debates over similarly disruptive technologies, such as genetic engineering or nuclear energy, did not result in such an extensive proliferation of subtopics and purported moral problems.

The second dimension can be characterized in terms of intensity, that is, cases in which the level of moral concern appears disproportionate to the actual risk. This issue has been noted in the literature. Danks and London (2017), for example, note that the much-discussed problem of “algorithmic bias” is often less morally problematic than commonly assumed and may even, depending on the goals pursued, be beneficial (moreover, the technical notion of bias seems to encourage a slide toward a moralized notion of bias or even prejudice). Powers and Ganascia (2020) place in this category debates over autonomous vehicles (particularly the claim that their deployment will generate unsolvable “trolley problems”), as well as concerns that deep neural networks might be trained to infer sensitive traits, such as sexual orientation, from facial images. Königs (2025) classifies as overrated both the use of deep learning systems in medical contexts—often criticized as opaque “black boxes” despite the potential primacy of diagnostic accuracy—and the frequently discussed “responsibility gap,” especially in relation to autonomous weapon systems, since it is unclear why those who authorize their deployment would thereby be absolved of responsibility for potentially harmful outcomes. The aim here is not to decide which issues in AI ethics are important and which are overrated, but rather to show that, on this question, there is skepticism even within the field itself.

The third dimension can be characterized in terms of misattribution (or misplaced moralization), that is, cases in which moral concern is projected either onto phenomena that are not, in themselves, genuine moral problems, or onto phenomena whose moral status is already well understood. On the one hand, many new technologies were known to cause so-called panic cycles, in which they were initially perceived as threats to established social and moral norms, only for such concerns to fade as societies adapted to them. Castro and McQuinn (2015) showed, for example, that technologies ranging from early portable cameras to more recent AI-related systems, such as drones and wearables, have typically undergone “privacy panic cycles” before becoming widely accepted and normalized. It would therefore not be unprecedented if at least some current concerns about AI reflected a similar tendency, with initial ethical concerns gradually subsiding as these technologies become more familiar and better understood. On the other hand, even when genuine moral concerns are present, they are often not novel in any meaningful or interesting sense. Consider the use of deepfake technology for blackmail or extortion: while AI enables the production of more convincing deceptive content, the underlying issue (deliberate deception for coercive purposes) is neither new nor ethically puzzling. As Müller (2022) aptly points out, for a problem to qualify as an AI ethics problem, it must be one for which we do not already know the right course of action.

A further indication of possible overmoralization can be found in how some prominent AI initiatives portray AI risks. For example, a series of documents published by the Future of Life Institute describes AI in alarmist terms. AI models are said to “pose major risks, such as the erosion of democratic processes, rampant bias and misinformation, and an arms race in autonomous weapons” (Future of Life Institute, 2026). A similar tone appears in the *Asilomar AI Principles*, which warn that advanced AI could represent “a profound change in the history of life on Earth” and pose “catastrophic or existential risks” (Future of Life Institute, 2017). *The Statement on Superintelligence* warns of “losses of freedom, civil liberties, dignity, and control,” and even “human extinction” (Future of Life Institute, 2025). The Center for AI Safety (2024) issued its *Statement on AI Risk*, stating that “mitigating the risk of extinction from AI should be a global priority alongside other societal-scale risks such as pandemics and nuclear war.” The open letter *Pause Giant AI Experiments* called for a global six-month moratorium on training systems more powerful than GPT-4 (Future of Life Institute, 2023). Although no such moratorium was implemented, the disastrous results that were predicted did not materialize.

These documents have been endorsed not only by AI experts but also by many philosophers and ethicists, including some of the most cited figures in the field. What is intriguing, however, is the following asymmetry: while there have been many critical responses from AI researchers warning against AI-related exaggerations and alarmism (Brooks, 2017; Mitchell, 2021; Stokel-Walker, 2024; Swoboda et al., 2026), comparable counter reactions (whether collective or individual) from philosophers and ethicists (such as emphasizing the benefits of AI development or the possible costs of unnecessarily slowing or restricting it) have remained almost nonexistent (cf. Königs, 2025). A more detailed analysis cannot be undertaken here, but it is worth noting that this stands in stark contrast to earlier ethical debates about similarly disruptive technologies. For example, the debate on human enhancement centered on whether it is permissible to use technological interventions—ranging from genetic to pharmacological—to enhance human cognitive, physical, or even moral capacities (Moseley and Juengst, 2025). In that context, positions among ethicists were relatively balanced, with both supporters and critics presenting well-developed arguments. In the case of AI ethics, however, the debate often seems one-sided, as if dominated by a single perspective focused mainly on identifying moral risks. While this asymmetry is far from conclusive, it may be interpreted as a further indication that AI ethics is, to some extent, shaped by factors beyond purely rational and methodological considerations, such as the evolved tendency toward overmoralization.

### 3.3 Overpropagating morally salient information

Why do claims about morally problematic aspects of AI spread so quickly? A key part of the answer may be a third cognitive mechanism identified by evolutionary psychologists: one that triggers increased attention to morally salient information, combined with highly effective ways of transmitting it. Human communication, from an evolutionary perspective, may have evolved largely to maintain group cohesion and coordination. Dunbar (1996, 2004) argued that language—particularly in the form of gossip—allows individuals to track reputational information, monitor norm adherence, and identify sources of social risk in ways that greatly surpass what direct interaction alone can achieve. Because of its survival and reproductive benefits, information signaling threats or socially relevant dangers is cognitively prioritized and systematically overpropagated. For this reason, narratives presented in amplified evaluative or moral terms (for example, those involving potential harm or injustice) are more likely to be noticed, remembered, and shared than those presented in more neutral terms. This tendency toward overpropagation can be further explained by using the memetic framework first proposed by Dawkins (1976) and developed by (Blackmore 1999; cf. also Aunger, 2000). According to this view, cultural evolution occurs through units of information called “memes,” which are acquired and transmitted through learning and imitation. Similar to genes in biological evolution, memes compete for replication, with their success not necessarily based on their truth or accuracy, but on their ability to be copied from one mind to another. Memes that are easy to remember and perceived as vitally important spread more effectively than those that are cognitively demanding or insignificant.

The hypothesis that such overpropagation may be occurring within AI ethics is consistent with the readily observable fact that contemporary research in applied ethics is dominated by projects, conferences, journals, books, and articles focused on AI. A further illustration of this pattern can be found in a basic search of the *Web of Science* database. As of March 2026, a search for publications containing the exact phrases “AI ethics,” “ethics of AI,” or “ethics of artificial intelligence” returned 3,268 results. While this number is modest compared with keywords and phrases related to long-established areas of applied ethics, such as “medical ethics” (19,765), “environmental justice” (13,094), “pornography” (8,681), or “affirmative action” (8,471), it is surprisingly high compared with other common topics, such as “animal rights” (2,823), “civil disobedience” (1,421), “conscientious objection” (1,419), “sexual ethics” (461), or “world hunger” (423). Two additional considerations, however, should be kept in mind in this context, which further suggest that AI ethics and its topics may be undergoing an unusual degree of overpropagation. First, its growth has occurred over a relatively short period: the earliest *Web of Science* record containing the phrase “AI ethics” is from 2015, whereas discussions of topics like medical ethics or affirmative action have developed for more than 50 years. Second, for simplicity, the above search focused only on the exact phrases “AI ethics,” “ethics of AI,” and “ethics of artificial intelligence”: if related (and nearly synonymous) phrases such as “machine ethics” and “robot ethics” were also included, the total number of relevant results would be much larger.

A similar pattern emerges when examining the growth of ethical discussions surrounding specific AI technologies. For example, *Web of Science* searches for literature on the ethics of “autonomous vehicles,” “autonomous cars,” and “self-driving cars” have returned 938 results since 2010, averaging about 59 per year. This starting point is not random: 2010 coincides with the public announcement of the Google Self-Driving Car project, which significantly boosted relevant research. In comparison, searches for “cloning ethics” or “ethics of cloning” have returned around 750 results since 1997 (about 25 annually), the year when the birth of Dolly the sheep was announced. As some authors note (Ori, 2020), there is a curious imbalance between the extensive literature on the ethics of autonomous vehicles (which remain far from large-scale adoption) and the relatively limited ethical focus on traditional road traffic, even though conventional traffic accidents cause more than one million deaths worldwide each year. Naturally, all these bibliometric data and their comparisons (taking into account differences in relevant technologies and their broader implications) should be interpreted with caution. Nevertheless, although alternative explanations for publication growth remain possible, the observed patterns are broadly consistent with the mechanisms outlined above.

An alternative way to address the overpropagation potential of certain topics in AI ethics could be to draw on what Dawkins (1976) and Blackmore (1999) have described as “religious memeplexes” (self-reinforcing groups of religious beliefs) and to consider whether it is meaningful to speak of emerging “AI memeplexes” (self-reinforcing groups of beliefs about AI). Clearly, religious beliefs and beliefs about AI are fundamentally disanalogous: the existence of various AI systems and their capabilities is empirically undeniable, while the existence of any deity remains unverifiable. The comparison drawn here concerns only the forms in which certain types of information are transmitted. It is difficult to avoid the impression that AI-related discourse often adopts forms highly similar to those characteristic of religious discourse. To highlight just a few possible parallels: just as religious memeplexes focus on an invisible, omnipresent, and all-knowing being influencing human life, AI stories increasingly portray AI as an all-pervasive, largely invisible infrastructure with superhuman cognitive abilities and an inevitable impact on human affairs; just as religions promise afterlife in heaven or hell, AI narratives suggest digital immortality through mind uploading or brain emulation; just as divine actions are described as inscrutable (“God moves in mysterious ways”), AI systems are often described as producing decisions that are opaque and hard to understand (“black-box” algorithms); and just as religious memeplexes contain prophecies of inevitable apocalypse or final judgment (all of which failed to materialize), AI narratives feature predictions of an inevitable singularity or AI takeover, accompanied by a similar record of unfulfilled forecasts (Boden, 2016). Again, the purpose of these parallels is not to equate AI narratives with religious ones, but to suggest that similar, evolved cognitive mechanisms—mechanisms that are highly effective at moralization, replication, and resisting rational argument—may operate in both domains.

“Bad is stronger than good” is the title and central idea of a study by Baumeister et al. (2001), which—drawing on a large body of psychological research on close relationships, social networks, interpersonal interactions, learning processes, and related domains—points precisely to what this phrase suggests: that negative events, information, and memories attract attention more quickly, are processed more thoroughly, and are remembered longer than positive ones. Although their goal was not to provide an ultimate explanation for this phenomenon, they suggest that it probably owes its existence to the evolutionary fact that organisms more attuned to negative stimuli have a better chance of survival and reproduction. AI ethics, as we have seen, is highly focused on the morally negative (“bad”) aspects of AI technologies and is pursued by descendants of organisms that were (one may plausibly assume) especially responsive to negative stimuli (organisms that were not responsive left no descendants). A reasonable hypothesis, therefore, is that an excess of negative information might also be present within this field.

## 4 Why AI ethics is particularly susceptible to the three triggers

The rapid expansion of AI ethics has been accompanied by a trend toward treating a wider-than-warranted range of AI-related developments as pressing moral problems. It was suggested in the previous section that much of this trend can plausibly be explained by the combined influence of three evolved cognitive mechanisms or triggers: the overdetection of agency, the overmoralization of affectively charged situations, and the overpropagation of morally salient information. Without specific empirical studies, the case presented in this paper regarding the possible impact of each trigger on AI ethics is necessarily indirect and circumstantial and should be understood as a conceptual framework comprising a set of arguments and explanatory hypotheses about a unique internal dynamics of AI ethics, with the potential to inform future empirical research and more precise, testable predictions. It should also be viewed as complementary to, but conceptually distinct from, typical accounts of AI-related hype (Cave and Dihal, 2019) and anthropomorphism (Li and Suh, 2022). The key difference can be articulated by drawing on two distinctions mentioned in Section 1. First, while AI hype and anthropomorphism are usually treated as relatively general and proximate psychological phenomena, the present approach is focused on three more specific cognitive mechanisms and their ultimate causes. Second, the three mechanisms most likely originated as adaptations to ancestral conditions—referred to in evolutionary psychology as the “environment of evolutionary adaptedness” (EEA)—which differed significantly from contemporary environments (Bennett, 2018). For this reason, when triggered in modern socio-technological contexts, their outputs become distorted in various ways. It is therefore plausible that the observed dynamics arise from a mismatch between cognitive mechanisms shaped by the EEA and the contemporary AI-saturated environments in which they are now applied. In what follows, three features specific to the field of AI ethics are examined that appear to amplify the activation of the three triggers (hereafter referred to as field-specific amplifying factors).

The first field-specific amplifying factor is the fact that many AI technologies and their ethical challenges are genuinely novel. AI development raises many profound questions across disciplines such as metaphysics and the philosophy of mind, including the nature of intelligence, action, representation, free will, creativity, and consciousness (Müller, 2025). Therefore, it may not be unreasonable for AI ethicists to follow suit and ask whether the technical opacity of certain algorithms prevents us from providing adequate justification for their decisions, or whether complex human-machine interactions create new types of responsibility gaps or dispersed accountability. Situations like these were largely absent from earlier phases of applied ethics, which focused mainly on moral issues involving humans, social institutions, and non-human animals, and it is understandable why they now attract increased ethical attention. The key point, however, is that the genuine novelty of some AI-related problems often spills over into neighboring problems that are not new, only slightly new, or only loosely connected to AI, leading to a persistent trend of treating an unusually broad range of AI-related developments as pressing moral problems. What further amplifies this pattern is that many experts in the technical community, themselves likely subject to wider cultural and extra-scientific influences (Cave and Dihal, 2019), often present AI innovations with considerable optimism. In response, AI ethicists—even when dealing with highly speculative or distant technological scenarios—often adopt a markedly critical or pessimistic stance, as if engaged in an evaluative arms race.

The second field-specific amplifying factor is the limited extent to which expert judgment, developed through specialized university education, protects against certain cognitive and moral biases. Drawing on a large body of empirical research, Kahneman (2011) famously argued that, especially under conditions of uncertainty, experts tend to be inconsistent in forming overall judgments from complex information and that their evaluations can be unstable and unreliable even when they possess substantial knowledge. For the present discussion, a particularly relevant empirical illustration (also emphasized by Kahneman) is Tetlock's (2005) long-term study of political and economic forecasting. In this study, several hundred experts were asked over many years to estimate probabilities for a substantial range of future political and economic events, both within and outside their areas of expertise, resulting in tens of thousands of individual predictions. The results showed that their forecasts were at best marginally better (and often no better) than chance-based predictions, and that increased expertise did not significantly improve accuracy. Moreover, experts displayed overconfidence and a reluctance to revise their views in light of contrary evidence, frequently rationalizing failed predictions after the fact.

A similar limitation can be observed in the domains of moral and philosophical judgment, and it is likely also present in moral evaluations of AI. This is demonstrated in several ways by the work of Eric Schwitzgebel and his colleagues, who are known for studies challenging the idea that philosophical training—especially in ethics—leads to more reliable moral judgment or behavior (Schwitzgebel, 2009; Schwitzgebel and Rust, 2009; Schwitzgebel et al., 2012). The most relevant finding for this discussion comes from their experiments on order effects in moral evaluation (Schwitzgebel and Cushman, 2012). Professional philosophers, non-philosopher academics, and lay participants were asked to evaluate familiar hypothetical cases involving well-known moral distinctions, such as the doctrine of double effect, action vs. omission, and moral luck. While the core content of the scenarios stayed the same, the order in which related cases were presented was systematically changed. The results were striking: philosophers (including ethics PhDs), just like other participants, changed their moral judgments depending on the sequence of scenarios. Schwitzgebel and Cushman (2012) concluded from this that philosophical training does not protect moral judgment from these biases and may even “enhance *post-hoc* rationalization,” noting that “professional philosophers' judgments about familiar types of cases in their own field can be strongly and covertly influenced by psychological factors that they would not endorse upon reflection.” If this diagnosis is correct, there is little reason to believe AI ethicists would be any more resistant to the kinds of biases generated by the evolved cognitive triggers discussed earlier.

The third field-specific amplifying factor can be linked to a form of professional self-interest, perhaps best captured by the well-known maxim “publish or perish.” This reflects a well-recognized structural feature of contemporary academic life: the ongoing pressure on researchers to produce a steady stream of publications in scholarly journals as a condition for securing employment, advancing their careers, and even retaining their positions within academia (Moosa, 2018). Smaldino and McElreath (2016) argue that this creates a Darwinian dynamic in which academic practices undergo a form of cultural “natural selection,” favoring research habits and methods that maximize publication output, often at the expense of rigor and truth. Davies and Felappi (2017) show that a similar predicament has not bypassed philosophy, arguing, among other things, that the pressure to publish quickly and in large quantities narrows the discipline by encouraging work on fashionable topics, since contributions to already popular areas are more likely to be recognized as valuable and published. Huemer (2023) offers a more precise diagnosis of ethics, noting that ethicists are especially motivated to continually identify new moral problems: since many of the “big” moral issues have already been thoroughly examined, it becomes hard to make truly novel contributions, which encourages presenting slightly harmful (or even harmless) phenomena as morally problematic.

These pressures seem to find particularly clear expression in the context of AI ethics. Königs (2025) provided an insightful account, arguing that the field is experiencing a “negativity crisis” (a systematic and unwarranted tendency to portray AI mainly in negative terms), which appears to align closely with the career incentives of its practitioners. Publishing, securing funding, and advancing professionally in AI ethics, according to Königs, are tightly linked to the identification of ever-new ethical problems. This is especially clear in large consortium projects involving computer scientists and engineers, where the ethicist's role often centers on identifying risks and flaws in the technology being developed. Concluding that a specific technology is ethically unproblematic, even if it is, is hardly career-enhancing, or, as Königs (2025) succinctly puts it, “ethicists who think that AI is ethically just fine are quickly out of business.” The underlying logic is straightforward: an academic environment that rewards professional survival and advancement by continually identifying new ethical concerns is likely to lower the threshold for activating the evolved cognitive tendencies discussed earlier. Although the precise psychological pathways involved would require further investigation, the underlying logic is straightforward: since the three triggers can be understood as mechanisms in the service of self-preservation and survival, it should not be surprising that they converge with academic incentives grounded in structurally similar forms of individual self-interest.

## 5 Conclusion

Why, then, do AI ethicists “shoot from the hip”? More specifically, why has AI ethics grown so rapidly alongside the tendency to interpret a wider-than-warranted range of AI-related developments as pressing moral problems? The answer proposed in this paper is that this can, to some extent, be understood as the result of three evolved cognitive mechanisms—overdetection of agency, overmoralization of affectively charged situations, and overpropagation of morally salient information—operating under conditions particularly conducive to their activation: the genuine ethical novelty of many AI technologies and the moral questions they raise, the limited capacity of ethical expertise to counteract certain cognitive and moral biases, and a professional environment that rewards self-interested academic behavior in the form of identifying ever-new moral problems.

How might one recognize that such “shooting from the hip”, or a shift from proportionate to disproportionate ethical concern, is taking place? The analysis suggests several indicators that may serve as red flags: a tendency toward strongly mentalistic or anthropomorphic descriptions of AI systems; sweeping generalizations that treat AI as a single phenomenon despite the diversity of its applications across markedly different domains; the treatment of familiar, longstanding, and even uncontroversial issues as if they were novel problems specific to AI; a strong focus on speculative—difficult to verify or falsify—technological scenarios as if they were imminent; and an unusual growth in publications and other scholarly activities—often predominantly negative—without corresponding conceptual or empirical novelty. Of course, none of these indicators is decisive on its own. Taken together, however, they may provide a rough guide to identifying where the three underlying cognitive triggers may be at work, while also helping to distinguish which concerns are more likely to be legitimate and which are overstated or otherwise insufficiently grounded.

The aim of this paper has not been to deny that AI raises ethical concerns, many of which are supported by evidence and recognized as important, but rather to explain why some concerns may proliferate and intensify, even if they do not correspond to the actual risks and capabilities of existing AI systems. It is also not suggested that AI ethicists consciously engage in overdetection of agency, overmoralization of affectively charged situations, or overpropagation of morally salient information. These processes operate largely outside conscious awareness, making their combined effects difficult to disentangle. Although each of the three cognitive mechanisms and the three field-specific amplifying factors discussed here could constitute a legitimate topic in its own right, they have been presented within a single conceptual framework that aims to offer a set of explanatory hypotheses about this specific internal dynamics of AI ethics and, hopefully, to pave the way for future empirical research and more precise, testable predictions.

If the considerations advanced here are on the right track, what broader implications might they have, and in which directions might they be further developed? For evolutionary psychologists, AI ethics and its growing community of practitioners may offer a particularly promising domain for testing and refining their theoretical models of specific cognitive mechanisms, including the mismatch between the conditions under which these mechanisms evolved and the contemporary socio-technological environments in which they are now deployed. For AI ethicists, engagement with evolutionary psychology could be equally beneficial, as it can help identify cognitive biases that may distort ethical judgment and, by increasing awareness of these biases, contribute to more nuanced and reliable assessments of AI technologies.
